# STK11 loss and SMARCB1 deficiency mutation in a dedifferentiated lung cancer patient present response to neo-adjuvant treatment with pembrolizumab and platinum doublet: A case report

**DOI:** 10.3389/fonc.2023.1088534

**Published:** 2023-01-27

**Authors:** Jianxin Chen, Junhui Wang

**Affiliations:** Department of Radiation Oncology, The Quzhou Affiliated Hospital of Wenzhou Medical University, Quzhou People′s Hospital, Quzhou, Zhejiang, China

**Keywords:** STK11, SMARCB1, lung cancer, pembrolizumab, efficacy

## Abstract

Cancers harboring serine threonine kinase (STK11) alteration or SWI/SNF-related, matrix-associated, actin-dependent regulator of chromatin, subfamily B, member 1 (SMARCB1) mutation are conventionally considered as treatment-refractory to immune checkpoint inhibitors or chemotherapy, respectively. However in the present report, we demonstrated a case of dedifferentiated non-small cell lung cancer, characterized by STK11 loss (due to promoter loss) mutation co-mutated with SMARCB1 deficiency mutation, has achieved significantly partial response to neo-adjuvant treatment with pembrolizumab and platinum doublet regimen. Our case highlighted that either STK11 loss, or SMARCB1 deficiency mutation, might not be used to select patients for PD-(L)1 blockade therapy or chemotherapy, respectively. SKT11 loss accompanied with SMARCB1 deficiency mutation may benefit from immunotherapy combined with chemotherapy.

## Introduction

Serine threonine kinase (STK11, also shown as liver kinase B1, LKB1) is considered as one of tumor suppressor genes, which encodes for a serine/threonine 11 that activates AMP-activated protein kinase (AMPK), hence being capable of regulating cell response to DNA damage, tumor growth, and energy metabolism (1, 2). There have been increasing studies reported that STK11 alterations be associated with primary resistance to immunotherapy agents including anti-programmed cell death protein 1 (anti-PD-1) and its ligand (anti-PD-L1) drugs, which have been emerged as standard first-line treatment with or without cytotoxic drugs (in the circumstance of PD-L1 expression ≥ 50%) in non-small cell lung cancer in absence of targetable mutations (3–8).

SWI/SNF-related, matrix-associated, actin-dependent regulator of chromatin, subfamily B, member 1 (SMARCB1, also known as integrase interactor 1, INI1) is also a tumor suppressor gene located in chromosome 22q11.2, expressed in the nucleus of almost all normal tissues of the body (9, 10). SMARCB1 is considered to be one of the core subunits of the chromatin remodeling complex SWI/SNF, a subfamily of ATP-dependent chromatin remodeling complexes functioned as involving in chromatin remodeling, dynamic modification of chromatin structure, changing chromatin from dense to open state, thereby controlling gene expression and regulating transcription mechanism proteins (11, 12). Mutation of SMARCB1 was frequently presented in sinonasal carcinoma, gastrointestinal cancer, and pancreatic cancer types, however, rarely reported in lung carcinoma (13, 14). According to the former literature, cancer patients with pathologic SMARCB1 mutations were reported resulting in a worse prognosis independently from the conventional treatment strategies including chemotherapy, radiation therapy or surgery (12, 15, 16).

Herein, we reported a rare case of dedifferentiated lung cancer harboring STK11 loss and SMARCB1 deficiency, due to p.S67X mutations, which presented nearly complete response to neo-adjuvant treatment with pembrolizumab and platinum doublet. Subsequently, he received radical left upper lobectomy, and still being disease free status.

## Case presentation

**Figure 1:**
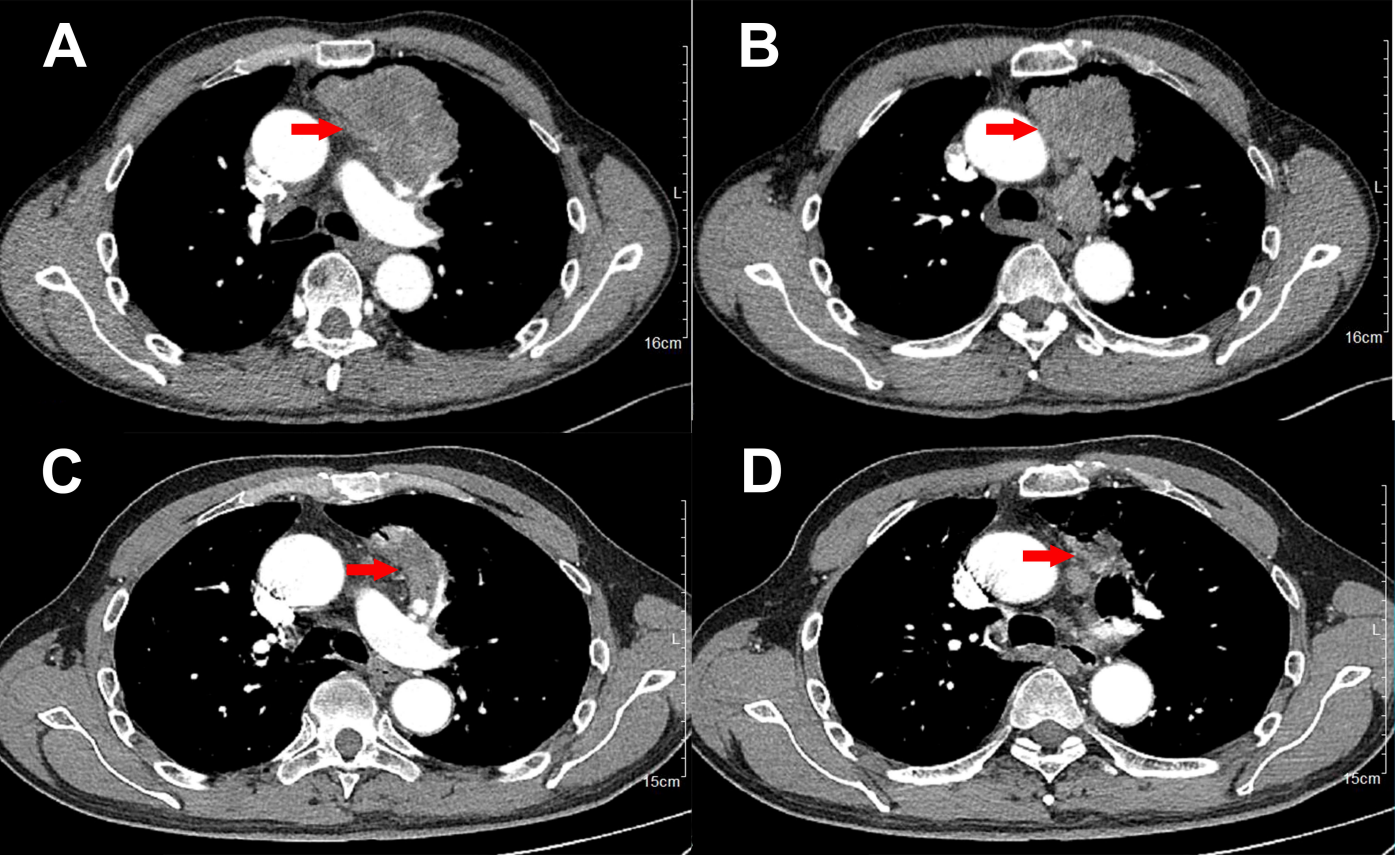
Variations of the primary lesions in lung by chest CT scans during the treatment (red arrowheads). **(A, B)**, June 6^th^, 2022. **(C, D)**, August 27^th^, 2022.

**Figure 2:**
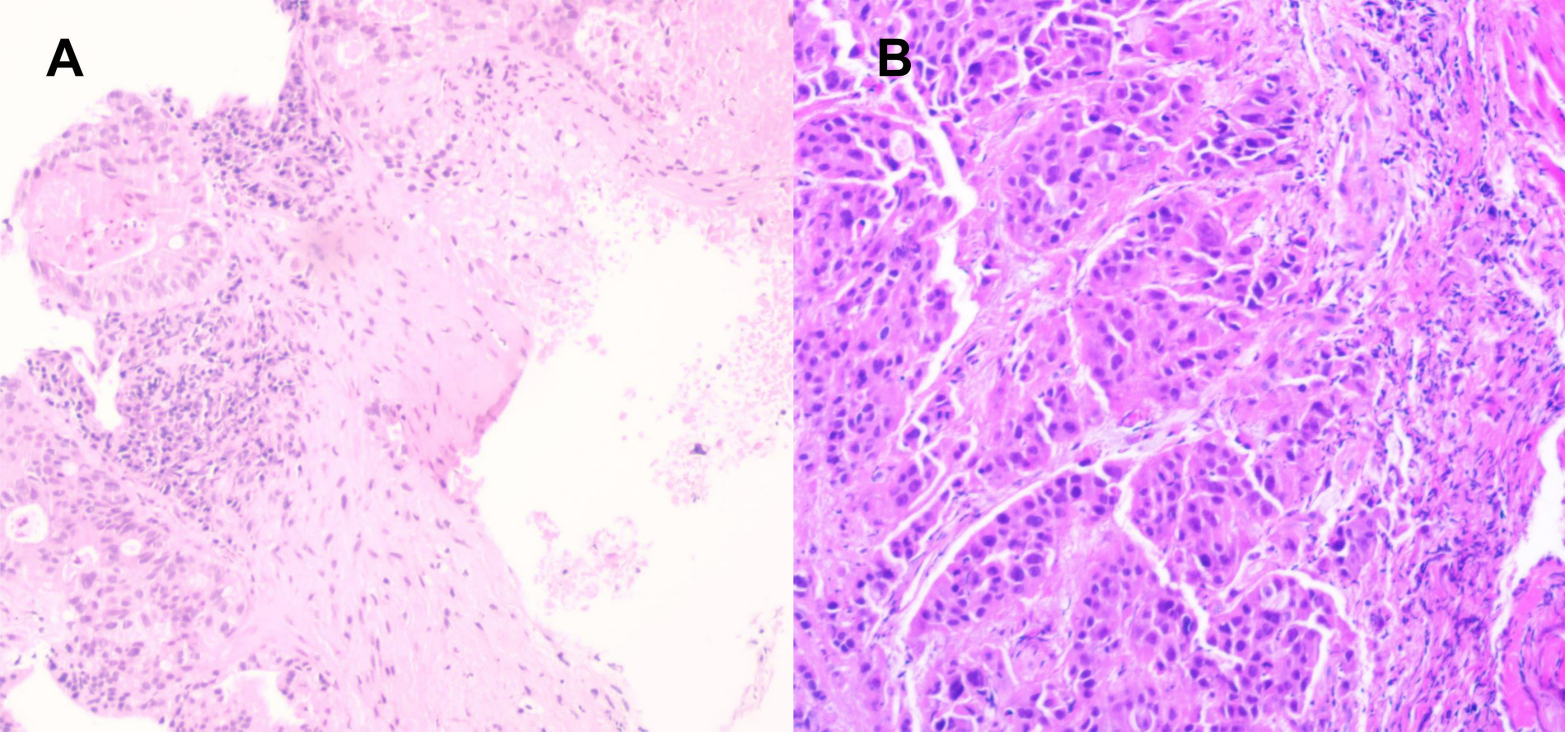
Histological findings with hematoxylin and eosin-stained biopsy specimen from biopsy on June 6^th^, 2022 (**A**, × 200), and surgery on September 6^th^, 2022 (**B**, × 400).

A 61-year-old Chinese man was admitted to hospital on June 6^th^, 2022 with chest distress and slight pain on left chest for approximately three months. He denied smoking or alcohol history, nor any other medical or family disease history. Chest CT on June 6^th^, 2022 showed a large space-occupying lesion (size as 8.8 × 5.1 cm) in the superior lobe of left lung, which was closed to mediastinum, presented as obstructive pneumonia, accompanied with vein and artery in left superior pulmonary involvement (**Figure 1A**). In addition, chest CT scan also revealed multiple enlargements of mediastinal lymph nodes (**Figure 1B**). Besides, there was no other distant metastatic lesion detected according to abdomen CT scans and brain MRI findings. Subsequently, he received CT guided percutaneous lung puncture biopsy, results of which revealed dedifferentiated carcinoma most likely originating from lung (**Figure 2A**) according to the imaging findings and immunohistochemistry outcomes presented as: TTF-1 (negative), NapsinA (negative), P40 (negative), P63 (negative), CK5/6 (negative), CK(AE1/AE3) (positive), Syn (negative), CgA (negative), CD56 (negative), INI-1 (negative), Ki-67 50% (positive), programmed cell death ligand-1 (PD-L1) negative. Furthermore, *via* next-generation sequencing (NGS) was performed using tissue sample (tumor cellularity 25%, including 550 cancer-related genes, BIOMED, China) showed STK11 loss, due to the loss of promoter (frequency as 30.67%) and SMARCB1 deficiency, due to p.S67X*10.74% mutations, with a tumor mutational burden (TMB) of 6.85 mutations per megabase, as well as PD-L1 tumor proportion score (22C3) being less than 1%. Based on those, the patient was diagnosed as dedifferentiated lung cancer, with vein and artery in left superior pulmonary involvement, along with metastasis on mediastinal lymph nodes, which was staged as IIIB (cT4N2M0, technically unresectable) according to the criteria of American Joint Committee on Cancer (AJCC) 8^th^ edition. After the discussion by multi-disciplinary team (MDT, including medical oncologist, surgical oncologist, radiation oncologist, and radiologist), regimen of chemotherapy combined with anti-PD-1 agent was adopted as palliative or neo-adjuvant treatment, which depended on the response status during the drug administration. In addition, maintenance therapy with anti-PD-1 agent should also be taken into consideration after disease control if necessary. Exactly, combined treatment with chemotherapy and immunotherapy has already been recommended as standard choice in advanced NSCLC patients in absence of sensitive targetable mutations. The patient thereby was started on pembrolizumab (200 mg on day 1, every 21 days), and carboplatin/nab-paclitaxel (nab-paclitaxel 260 mg/m^2^ on day 1, carboplatin AUC 5 on day 1, every 21 days) as initial treatment. After three cycles′ treatment, restaging chest CT scans showed significant improvement on the primary lesion, as well as metastatic mediastinal lymph nodes (**Figures 1C, D**). Repeated brain MRI and abdomen CT scans did not suggest any progression disease on other organs either. Efficacy assessment was evaluated as partial response (PR) according to the criteria of Response Evaluation Criteria in Solid Tumors (RECIST) version 1.1. Restaging by the above imaging assessment was resulted in down-staging as cT2N2M0 (IIIA). Based on the downstaging obtained, the patient underwent a radical left upper lobectomy on September 6^th^, 2022. Pathologic findings from the surgery confirmed the diagnosis of dedifferentiated lung carcinoma (**Figure 2B**). Immunohistochemistry outcomes were similar to the original biopsy with evidence of immune cell infiltration observed. On October 4^th^, 2022, the patient switched to maintenance therapy with pembrolizumab four weeks after the surgery. The only adverse event observed during the treatment was neutropenia (grade 2 according to Common Terminology Criteria Adverse Events version 5.0), which happened after the first cycle exposure of the combined regimen. At the time of manuscript preparation, the patient continues to receive mono-therapy of pembrolizumab, and still maintained an ongoing disease free status.

## Discussion

Herein in the present report, we demonstrated a case of dedifferentiated non-small cell lung cancer, characterized by STK11 loss and SMARCB1 deficiency, due to p.S67X mutations, which has been conventionally considered as treatment-refractory, has achieved significantly partial response to neo-adjuvant treatment with pembrolizumab and platinum doublet regimen. To the best of our knowledge, the present case was the first one to report the co-alteration of STK11 loss and SMARCB1 deficiency mutation. Results of the present case highlighted that biomarkers including loss, or SMARCB1 deficiency mutation, might be probably predictive to response to PD-1 blockade therapy.

Somatic STK11 mutations were reported presenting in approximately 30% of non-small cell lung cancer patients, especially among smokers (17). In addition, STK11 mutation often occurred with co-mutation with kirsten ratsarcoma viral oncogene homolog (KRAS) and kelch-like ECH-associated protein 1 (KEAP1), which usually resulted in even worse prognosis (18, 19). Most recently, a retrospective study was conducted among USA institutes including the Dana-Farber Cancer Institute/Massachusetts General Hospital, the Memorial Sloan Kettering Cancer Center, and the MD Anderson Cancer Center, to investigate the efficacy prediction role of STK11/KRAS/KEAP1 mutations to immunotherapy efficacy (4). Results of the observational study showed that STK11 and KEAP1 mutations were associated with significantly worse PFS (STK11 hazard ratio (HR) = 2.04, *P* < 0.0001; KEAP1 HR = 2.05, *P* < 0.0001) and OS (STK11 HR = 2.09, *P* < 0.0001; KEAP1 HR = 2.24, *P* < 0.0001) survival to immunotherapy among KRAS mutant lung adenocarcinoma (4). Besides, another retrospective research also drew the similar conclusion. CLICaP was a retrospective study performed to evaluate a cohort of Hispanic patients diagnosed with metastatic NSCLC from the US and seven Latin American countries treated with immune checkpoint inhibitors (ICIs) alone or in combination as first-line treatment. Results of the study revealed that OS was significantly shorter for patients carrying STK11 mutations (STK11-mutant 14.2 months versus STK11-wild 27.0 months (*P* = 0.0001)) or KEAP1 (KEAP1-mutant 12.0 months versus KEAP1-wild 24.4 months (*P* = 0.005)) mutations (3). Although the potential mechanism still remained uncovered, STK11 has been proposed to induce T-cell exhaustion and immune-suppressed or “cold” tumor microenvironment according to relevant literatures (20). Nevertheless, there were studies reported that STK11 mutation may be associated with inferior outcomes by combined platinum-doublet plus immunotherapy even in high PD-L1 expression and high TMB patients (21, 22). However in the present case, the mutant type of STK11 was presented as the loss promoter of STK11 gene, which may lead to the silence expression of STK11 gene, thereby resulted in function deficiency of tumor suppressor. The difference of the alteration types might stand as one of potential reasons for the diverse clinical outcomes. Besides, the missence of SMARCB1 p.S67X*10.74% mutation may also contribute to the favorable outcomes in the present case, prognosis of which was consistent to a recent study as mentioned before (16).

SMARCB1-deficient carcinoma is a recently recognized entity in several organs, especially reported in sinonasal carcinoma (13, 23). Mutation of SMARCB1 deficiency was rarely reported in thoracic neoplasms. It was not until 2018 that Yoshida et al. reported the first case of SMARCB1-deficient pleural squamous cell carcinoma (24). In that case, patient with SMARCB1-deficient carcinoma of the pleura represented primary resistant to cisplatin plus gemcitabine therapy, thereby progressed rapidly and finally resulted in 10 months OS after diagnosis (24). Coincidentally, another recent case also reported the characteristics of poor prognosis and chemotherapy resistance in a Chinese patient with lung cancer harboring SMARCB1 deficiency, of which OS presented as 5 months merely (25). However, there were several studies with limited sample suggested that immunotherapy, including immune checkpoint inhibitors, may present as a promising therapeutic strategy for SMARCB1-deficient tumors, especially in sarcomas, and sinonasal carcinoma (26, 27). Mechanistically, SMARCB1 deficiency favored the de-repression of multiple endogenous retroviral elements (ERVs), resulting in cytosolic double-stranded RNA accumulation which activates cytoplasmic sensors including TLR3 and MDA5, and thereby cell-autonomous IFN-α and IFN-λ responses (27). In addition, an improved anti-cancer innate immune response driven by ERVs de-repression has also been observed in various cancer types (28), and correlated to response to anti-PD-(L)1 treatment (29). Practically in the present case, the favorable outcomes of the patient by immunotherapy also suggested that anti-PD-(L)1 treatment might be potentially effective in non-small cell lung cancer patients harboring SMARCB1 deficiency. Comparing to the former results by those published literatures, we speculated that the emerging mutation of STK11 loss (due to the loss of promoter) may play a role on the different outcomes between the present case and former studies. The co-current mutation of STK11 loss and SMARCB1 deficiency may offset the aggressive outcomes by SMARCB1 deficiency, hence resulted in the sensitivity of chemotherapy, as well as anti-PD-1 inhibitors. Even so, further investigation for potential mechanism by basic experiments should still be needed. In addition, because of the limited sample size in the present case report, a larger sample size in real world status should also be necessary to identify the clinical phenomenon.

Despite numbers of literatures suggesting the biological plausibility of SKT11 mutation may lead to primary resistant to anti-PD-(L)1 therapy, the issue is far from settled. An exploratory analysis from the trial KEYNOTE-042 revealed that pembrolizumab monotherapy was associated with improved OS when compared with platinum-doublet regardless of STK11 and KEAP1 mutational status (30). Just be similar to that, we have reported that SKT11 loss co-mutation with SMARCB1 deficiency mutation may also benefit from immunotherapy combined with chemotherapy. Recently, an enhancer of zeste homolog 2 (EZH2) inhibitor tazemetostat, has been approved by Food and Drug Administration (FDA) for the treatment of SMARCB1-deficient epithelioid sarcoma, representing the first molecularly targeted agent for SWI/SNF-deficient malignancies (31). However, the effectiveness of the target agent on NSCLC patients has not been established yet, which need further identification. Therefore, the combined regimen adopted in the present case might prove some therapeutic thoughts for clinical physicians.

## Conclusion

In the present report, we demonstrated a case of dedifferentiated non-small cell lung cancer, characterized by STK11 loss and SMARCB1 deficiency mutation that conventionally been considered as treatment-refractory, has achieved significantly partial response to neo-adjuvant treatment with pembrolizumab and platinum doublet regimen. Our case highlighted that either STK11 loss, or SMARCB1 deficiency mutation, might not be used to select patients for PD-(L)1 blockade therapy or chemotherapy, respectively. In addition, awareness and recognition of the co-mutations of STK11 loss and SMARCB1 deficiency mutation may help sharpen focus on effective therapeutic development, and expand our understanding on the potential mechanism, as well as pathological significance of the rare co-mutations.

## Data availability statement

The original contributions presented in the study are included in the article/supplementary material. Further inquiries can be directed to the corresponding author.

## Ethics statement

The studies involving human participants were reviewed and approved by Ethical Committee of People′s Hospital of Quzhou. The patients/participants provided their written informed consent to participate in this study.

## Author contributions

JC, conceptualization, methodology, software, writing- original draft preparation, software, and validation. JW, data curation, visualization, investigation, supervision, writing-reviewing, and editing. All authors contributed to the article and approved the submitted version.
